# Flapless Surgical Approach to Extract Impacted Inferior Third Molars: A Retrospective Clinical Study

**DOI:** 10.3390/jcm10040593

**Published:** 2021-02-04

**Authors:** Alberto Materni, Nicola De Angelis, Nicolò Di Tullio, Esteban Colombo, Stefano Benedicenti, Andrea Amaroli

**Affiliations:** 1 Department of Surgical Sciences and Integrated Diagnostic (DISC), University of Genova, 16132 Genova, Italy; materni@maternipiano.it (A.M.); n.deangelis74@gmail.com (N.D.A.); esteban.colombo92@gmail.com (E.C.); stefano.benedicenti@unige.it (S.B.); 2 Department of Health Science (DISSAL), University of Genova, 16132 Genova, Italy; nicolo.ditullio@gmail.com; 3 Faculty of Dentistry, Department of Orthopaedic Dentistry, First Moscow State Medical University (Sechenov University), 119991 Moscow, Russia

**Keywords:** third molar extraction, third molar surgery, impacted tooth, oral surgery, mandibular molar, flap design

## Abstract

This study aimed to compare a flapless surgical approach (FSA) with a traditional envelope flap (traditional approach (TA)). Every patient was treated with two approaches: TA and FSA. The primary outcome variables were both the discomfort during the post-operative convalescence and the correct final recovery of the impacted area. The secondary outcome variable was the average duration of the surgery. Post-operative pain and oedema were recorded. The measurements of soft tissue interface toward the distobuccal edge of the second molar were taken by periodontal probe before surgery (baseline) and 8 weeks after surgery. Statistical software was used to evaluate the data; a *p*-value < 0.05 was considered statistically significant. Twenty-four teeth of 12 patients (six Caucasian males and six Caucasian females, aged 23 ± 4 (17–30) years) with both lower impacted third molars (Ms3) were analysed. Considering an alpha error 0.05 that sample size allows power from 0.80 to 0.90, depending on the variable evaluated. Concerning attached gingiva, oedema and pain, the linear mixed model resulted in a statistically significant difference between the TA and FSA (*p* = 0.003; *p* < 0.01; and *p* = 0.018, respectively). Conversely, the model did not show a difference (*p* = 0.322) if pocket probing depth was considered. The FSA procedure was faster (*p* < 0.05) than the TA procedure (17 min and 8 s (±6 s) vs. 28 min and 6 s (±4 s), respectively). The results suggest that the FSA could be a suitable option for improving the surgical removal of lower Ms3. However, additional randomized controlled trial studies are necessary to confirm the reliability of our procedure and to verify its suitability in more complex Ms3 classifications.

## 1. Introduction

Removal of impacted third molars (Ms3) is one of the most common surgical interventions performed in dentistry [1]. In Norway alone, about 75,000 Ms3 are removed annually, often by oral surgeons [2,3]. In fact, impacted Ms3 can require a removal procedure that frequently consists of a surgical flap, ostectomy and odontotomy (of the tooth), as well as soft tissue suturing [4]. The manipulation of both soft and hard tissues can, unfortunately, cause post-surgical swelling and pain, reduced masticatory ability, trismus and periodontal diseases at the distal aspect of the mandibular second molar (M2) [1,4,5,6,7]. Concerning the surgery employed, it is evident that there are conflicting results on the effectiveness of surgical M3 removal, with varying outcomes in the adjacent M2 [5]. Many researchers have examined the effects of mucoperiosteal flap designs, such as the envelope flap, triangular flap, Szmyd flap and modified versions of these flaps. Suarez-Cunqueiro et al. [8] suggested that the envelope flap and triangular flap are the most widely used techniques for the removal of impacted Ms3. In this context, a careful literature review by Alqahtani et al. [5] concluded that the envelope flap design is better than the triangular flap when discomfort is considered, while the triangular flap is more efficacious for dehiscent wound healing and the post-operative periodontal status of the adjacent M2. However, studies have revealed that the Szmyd and paramarginal flap designs may be most effective for periodontal healing after impacted M3 extraction [4]. Moreover, studies highlight that surgical drainage has a positive effect on postoperative reactions after the removal of an M3 and represents an alternative for the perioperative management of impacted M3 surgery [6].

Nowadays, various standards have been proposed, but the literature shows that there are conflicting data on the effect of the surgical removal of Ms3, with varying outcomes for post-operative re-injury [5]. Within this context, it is clear how the optimal management of impacted Ms3 continues to challenge clinicians [9].

One of the most common adverse events after the surgery is swelling, accompanied by various degrees of pain. The most accredited hypothesis for this side effect can be mainly attributed to the full-thickness flap elevation and the extent of bone removal to expose and remove the retained tooth. The novelty of our work is purposing a flapless surgical approach (FSA) for lower M3 removal. The investigators hypothesize that, according to the literature on dental implant placement, a flapless approach would probably have less associated post-operative discomfort when used in the lower M3 removal [10,11,12]. The specific aims of the study were to retrospectively analyse the results of FSA with the traditional envelope flap (also referred as the traditional approach (TA)) in terms of pain, oedema, attached gingiva, and pocket probing depth recovery, as well as the average duration of the surgery. The null hypothesis was that there is no difference between the novel FSA and the TA. The clinical importance of the study is that a reduction of side effects and the speeding of the surgical approach improve the patient’s discomfort during and after the operation.

## 2. Materials and Methods

This study followed the Declaration of Helsinki with regard to medical protocol and ethics, and the Regional Ethical Review Board of DISC, University of Genoa approved the study (Unige-Disc-protocol number 0032792/23-06-2018). A written informed consent was obtained from all patients, who voluntarily agreed to undergo the procedure and declared their willingness to return at regular intervals for evaluation. Signed patient releases were also obtained for the use of images. The study population was composed of all patients that showed up for evaluation of extraction of lower Ms3. The experimental study was managed at the Department of Surgical and Diagnostic Sciences (DISC), University of Genoa, (Genoa, Italy). Patient recruitment and follow-up were completed between January 2018 and September 2018. To be included in the study sample, patients had to meet the following inclusion criteria: (1) both lower Ms3 compromised with a similar situation (left and right); namely, vertical or mesioangular in accordance with the Winter classification [13] and class I-II and A- B according to the Pell and Gregory classification [13] and (2) age 15–35 years. Patients were excluded as a study subject for the following: (1) autoimmune diseases; (2) diabetes; (3) current smoking; (4) pregnancy; (5) chronic disease and systematic drug therapies; (6) stomatitis and, in general, patients with a significant medical condition; and (7) full-mouth plaque score (FMPS) > 20%; please see Figure S1. Before scheduling the patient for the surgery, adequate professional hygiene and a review of oral maintenance were performed to achieve optimal plaque control with an average F.M.P.S. < 20% in the study population. The participants were also radiologically screened with an orthopanoramic X-ray (Planmeca ProMax^®^ with one-shot cephalostat, Helsinki, Finland). The pre-operative surgical evaluation was performed according to the Winter and Pell and Gregory classifications [13]. The classification was performed to determine the probable difficulty of extraction of both Ms3. As described in the inclusion criteria, only patients with a similar situation (left and right) were accepted. In case of overlap of the tooth with the inferior alveolar nerve on the orthopantomogram (OPT), cone-beam computed tomography (CBCT) (Planmeca ProFace^®^, Helsinki, Finland) was requested in order to clarify the three-dimensional anatomy of the overlap. All the surgeries were done by the same oral surgeon (A.M.) and the both the teeth of the patient extracted on the same day. The sequence, left and right, of the teeth extracted and the technique employed, FSA or TA, were randomly performed by draw. The patients were not informed about the approaches made. The number of participants (12 patients), as well as the number of total teeth extracted (24 teeth), were in accordance with Monaco and co-authors [14] as well as the sample size power calculation according to preliminary data. Basically, considering the preliminary mean value of two primary variables such as attached gingiva and oedema (mu 3.5–2.9; sigma 0.7–0.6), a sample size of 12 patients is necessary to have a power of 80% with an alfa of 0.5. A mouth rinse of 1 min with 0.20% chlorhexidine was done at the beginning of all surgeries. Lower alveolar nerve block anaesthesia was provided through the injection of 1.8 mL of articaine [15] with adrenaline 1:100,000 bilaterally (Pierrel S.p.A., Milan, Italy). Peripheric local anaesthesia was then performed all around the wisdom tooth positions, extended buccally and lingually forward to the first molars using another ca. 1.5 × 1.8 mL articaine with adrenaline 1:100,000 per side (total 2.5 × 1.8 mL per side; 5 × 1.8 mL per patient). An intramuscular injection of 8 mg of dexamethasone [16] (Laboratorio Farmacologico Milanese, Varese, Italy) was then provided to all patients just before the beginning of the surgical extractions in the masseteric region, dividing the 8 mg/2 mL ca. 50% per side.

### 2.1. Study Variable

The predictor variable was the type of incision design. The primary outcome variables were the discomfort during the post-operative convalescence and the correct final recovery of the impacted area. The secondary outcome variable was the average duration of the surgery. Primary outcome measures, such as post-operative pain, discomfort, and oedema were recorded with the Visual Analogue Scale (VAS) in the first week after surgery (days 1, 2, and 3); all participants were requested to assign a score for each side, as well as the number and timing of medications taken. Clinical data were also recorded with particular attention to the soft tissue interface toward the distobuccal side of the 2M, exhibiting the extent of operative gingival recession. Therefore, the additional primary outcome measure was taken by a periodontal probe (Hu-Friedy CP UNC 15, Chicago, IL, USA) before surgery (baseline) and 8 weeks after surgery and expressed in mm. To avoid bias, according to Monaco et al. [14], all measures were performed in triplicate by three authors with surgeon experience. The secondary outcome measure, the duration of the intervention, was measured using an iPhone stopwatch (Apple, Cupertino, CA, USA).

### 2.2. Surgical Design

**Figure 1:**
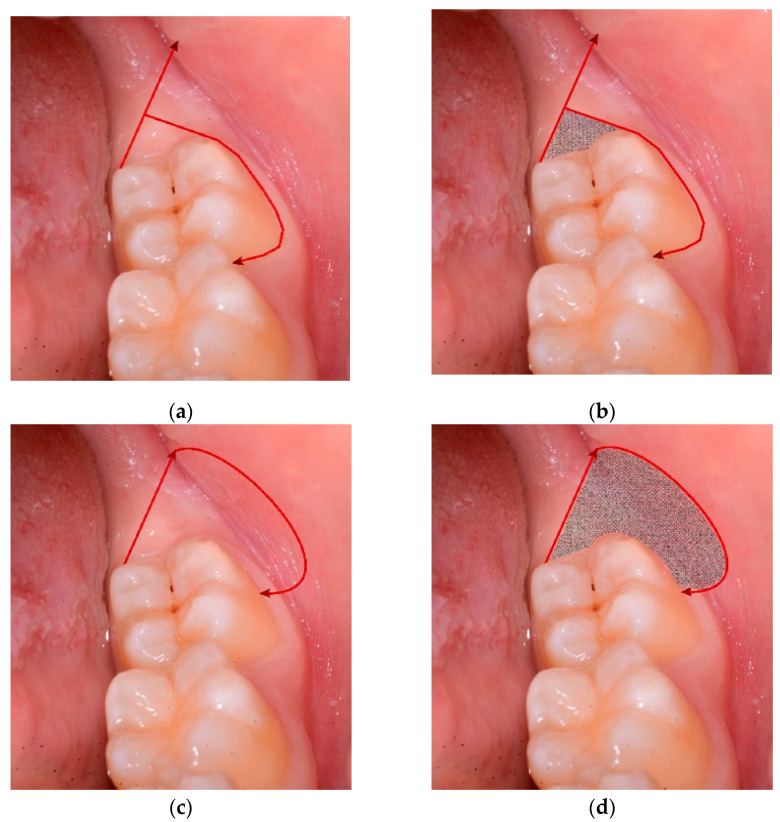
This figures explain the design of the surgical incision: (**a**,**b**) traditional envelope flap (traditional approach, TA); (**c**,**d**) the novel access design (Flapless surgical approach [FSA]). The area of partial thickness tissue removed is highlighted in grey (**b**–**d**).

Traditional approach (TA): The traditional envelope flap consists of a first distal incision beginning from the distal surface of the 2M in the attached gingiva and moving the 15c Swann-Morton blade distobuccally 45° for around 10 mm. The starting point must be in the attached gingiva to avoid lingual nerve damage. Therefore, the beginning of the incision can be at the distolingual cusp of the 2M or shifted to the distobuccal cusp in case of the absence of attached gingiva in that area. The second incision began perpendicularly from the first incision around 3–4 mm distally from the 2M, directed to the buccal sulcus of the 2M, going through it and ending in the buccal papilla between the second and first molars (Figure 1a). A soft tissue triangle was defined just distal to the 2M, which was then eliminated with the help of a mini-Friedmann 90° rongeur (RMF90 rongeur Friedmann 90°, small cod RMF90, Hu-Friedy Mfg. Co., Chicago, IL, USA) (Figure 1b). By using a Prichard periosteal elevator (3 Prichard periosteal PPR36, Hu-Friedy Mfg. Co., Chicago, IL, USA), a full-thickness flap was then elevated, exposing the buccal and also the occlusal bone in the case of fully impacted wisdom teeth to the oblique mandibular line. The flap was protected on the buccal side with an 11 × 40 mm Langenbeck metal retractor (Stoma Dentalsysteme GmbH & Co KG, Emmingen-Liptingen, Germany).

**Figure 2:**
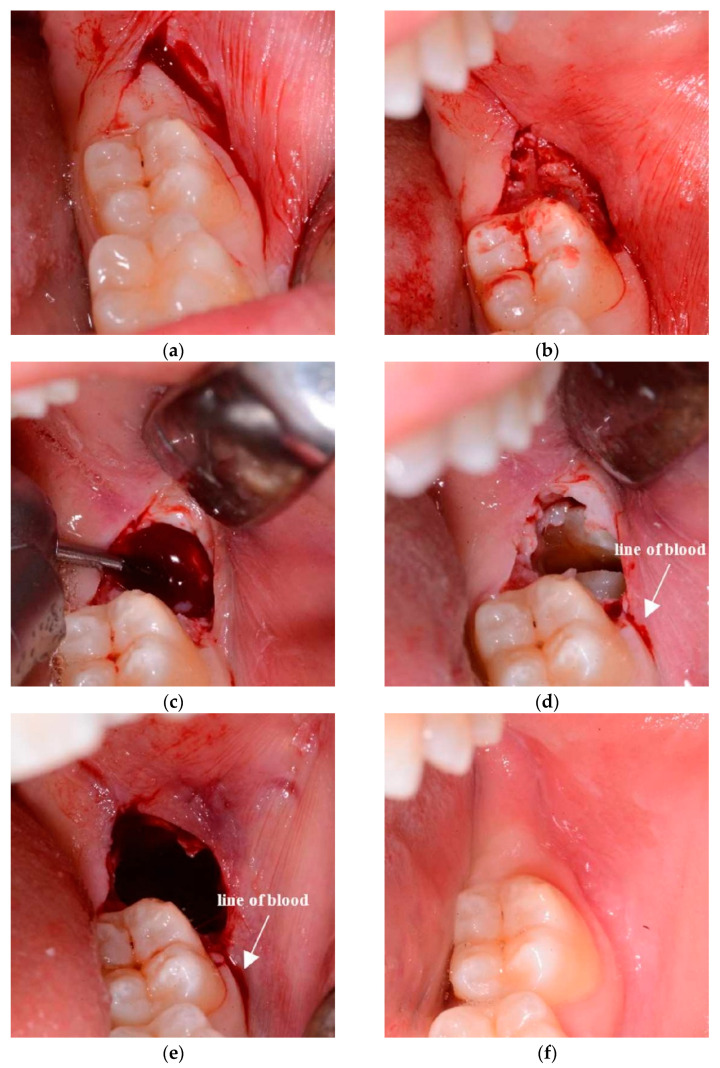
Images of the surgery with the flapless surgical approach (**a**–**e**) and wound healing of post-operative recovery (**f**). (**a**) Area of soft tissue delimited by 2 incisions; (**b**) partial thickness tissue area removed; (**c**) ostectomy; (**d**) odontotomy; (**e**) end of the surgery: the wound heals through an open follow-up core, so sutures are absent. (**f**) At 8 weeks, the wound is healed and the soft tissue physiologically recovered.

Flapless surgical approach (FSA): consists of a first distal incision equal to that on the control side (TA). The incision begins from the distal surface of the 2M in the attached gingiva, moving the 15c Swann-Morton blade distobuccally 45° (Swann-Morton Limited Owlerton Green, Sheffield, UK) for around 10 mm. The starting point must be in the attached gingiva to avoid lingual nerve damage. Therefore, the beginning of the incision can be at the distolingual cusp of the 2M or shifted to the distobuccal cusp in case of the absence of attached gingiva in that area. The second incision begins at the distal end of the first incision and describes approximately the form and position of the probable ostectomy, which has to be performed against the buccal surface of the impacted wisdom crown. The blade describes herewith a semicircle in the buccal mucosa, ending against the buccal surface of the 2M in correspondence of the enamel sulcus, which divides the two buccal cuspids (Figure 1c and Figure 2a). The area of soft tissue is now delimited from the two incisions and the surface of the M2. The mucosa distal to the M2 is removed (Figure 1d and Figure 2b) with the help of a mini-Friedmann 90° rongeur, so a kind of partial-thickness flap is realized just above the impacted M3.

In both cases, a buccal ostectomy is performed using a surgical steel bur (Komet H31LR316 016, Komet Dental Gebr. Brasseler GmbH & Co., Lemgo, Germany) in a 45° angle surgical handpiece with cooling water port and no air spray (NSK TI-MAX 45° Stand-Titan, NSK Dental, Kanuma, Japan) (Figure 2c). The use of a traditional air-driven motor to ablate soft tissue is not recommended, because its features might cause tissue emphysema. Additionally, the only soft tissue of the buccal side has to be removed to avoid lingual nerve damage. Basically, the only difference between the two sides is that, on the test side, the bur cuts not only the bone but also the overlying partial-thickness soft tissues, such as the periosteum and part of the remaining connective tissue.

An odontotomy (Figure 2d) is then performed in order to be able to extract the tooth more efficiently through the use of surgical steel bur (Komet H31LR316 016, Komet Dental Gebr. Brasseler GmbH & Co., Lemgo, Germany) in a 45° angle surgical handpiece with cooling water port and no air spray (NSK TI-MAX 45° Stand-Titan, NSK Dental, Kanuma, Japan).

The post-extraction socket is then controlled with the use of a 3 mm Lucas bone curette in the superficial part, without putting the metal instrument deep into the apical portion of the alveolus in order to not create unintentional damage to the inferior alveolar nerves. The side treated by the TA ends with a monofilament synthetic absorbable poliglecaprone 4/0 USP surgical PGCL suture with a 16 mm 3/8 OMNIA reverse cutting needle (OMNIA S.p.A., Fidenza, Italy), which ensures the flap stays in its original position, without tension, leaving triangular open communication distal to the 2M to achieve an open healing process. A knot is made to keep the flap distal to the 2M in an apical position, and normally two other single knots are made to keep the distal incision closed.

The side treated with the FSA ends without suture (Figure 2e) because no flap has been elevated and no soft tissue has free mobility. The alveolus on the test side surgery is left to heal naturally without external help, just like a normal post-extraction socket of an erupted tooth. Every patient was discharged after being instructed with the post-surgical indications as follows: (1) ice in contact with the cheek, alternating the side every 5 min all day long (2) only cold food for the first day and no rinsing for the first day, (3) take the prescribed ibuprofen 600 mg, 1/12 h–3 days (Ibuprofen Sandoz, Sandoz-Novartis, Holzkirchen, Germany), (4) mouth rinse with 0.12% chlorhexidine from the day after surgery, after eating, for the whole week. No antibiotic therapy was administered, according to previously used protocols [17,18,19].

### 2.3. Statistical Analysis

Primary and secondary outcome measures were statistically analysed. The mean ± standard deviation of measures and scores data, as well as the statistical power of the sample size, were taken into account. In the assessment of the data, a linear mixed model was used. Calculations were performed using the SPPS 25 (IBM Corp. Released in 2017. IBM SPSS Statistics for Windows, Version 25.0, Armonk, New York, NY, USA) statistics package program. For the significance level of the tests, the value of *p* < 0.05 was accepted.

Sample size was calculated by MedCalc Statistical Software version 16.4.3 (MedCalc Software, Ostend, Belgium).

## 3. Results

### 3.1. Patient and Baseline Characteristics

The retropective analysis was conducted on 24 teeth of 12 patients with both lower Ms3 impacted (please see Figure S1). The patient population was composed of six Caucasian males and six Caucasian females, aged 23 ± 4 (17–30) years. According to the exclusion criteria, the patients having compromised Ms3 in a similar situation at the left and right sides; namely, vertical or mesioangular in accordance with the Winter classification and class I-II and A-B according to the Pell and Gregory classification. Five patients showed I-B classification, four patients I-A and three were II-B.

Patient recruitment and follow- up were completed between January 2018 and September 2018.

### 3.2. Clinical Follow-Up and Outcome Measures

Concerning the attached gingiva, patients experienced a worsening in 17% of cases for TA and 8% for FSA, while concerning the pocket probing depth, only one patient treated with the FSA had problems (and his probing depth got worse). No other major important harms or unintended effects in each group were experienced. As reported in Table 1, in accordance with the analysis on attached gingiva (AG) and pocket probing depth (PPD), the description of recorded variables split between the TA and FSA expressed as mean (SD) were:

Traditional envelope flap (TA) (12 Ms3) = AG baseline 3.4 mm (0.6 mm)—AG 8 weeks 3.2 mm (0.8 mm); PPD baseline 5.4 mm (1.9 mm)—PPD 8 weeks 3.5 mm (1.0 mm). Flapless surgical approach (FSA) (12 Ms3) = AG baseline 2.9 mm (0.8 mm)—AG 8 weeks 3.3 mm (0.5 mm); PPD baseline 5.4 mm (2.1 mm)—PPD 8 weeks 3.6 mm (1.0 mm).

**Figure 3:**
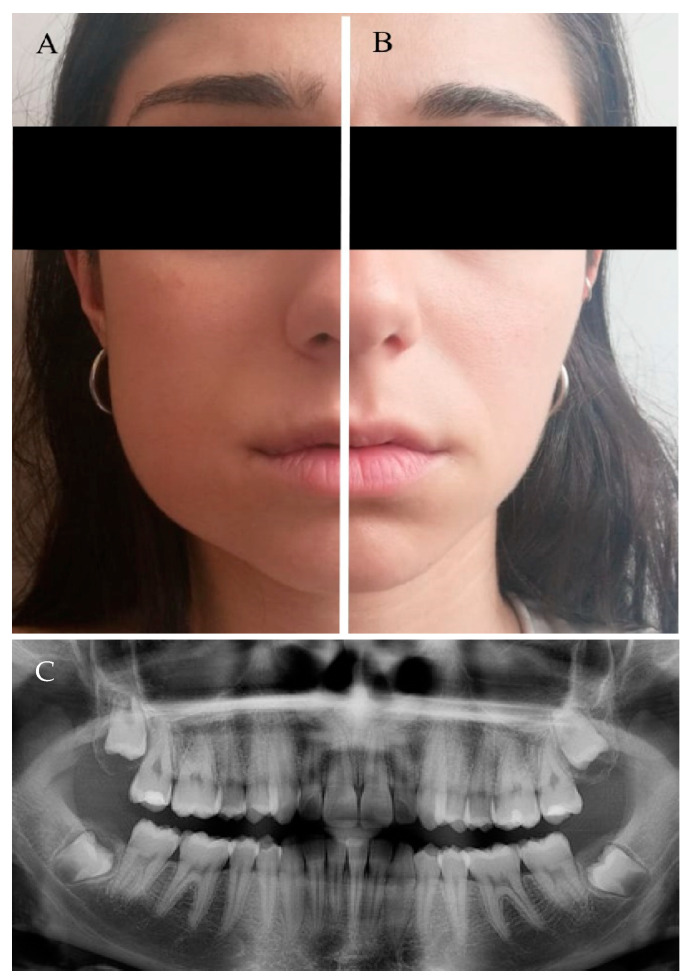
Every patient was randomly treated, on the same day, by both approaches: traditional envelope flap (TA) and our flapless surgical approach (FSA). The image shows a patient with a evident oedema on the TA side (**A**), while oedema is not present on the FSA side (**B**). (**C**) panoramic X-ray of the patient’s teeth.

No oedema symptoms were detected 24 h after the surgical removal of Ms3 for either procedure. Conversely, the difference was observed 48 h after surgery between the TA and FSA, and the differences increased at 72 h (Table 2 and Figure 3). Similarly, by comparing the two techniques, no differences concerning the pain were observed 24 h after surgery (*p* > 0.05). However, at 48 h and 72 h, patients experienced a significantly higher level of pain from surgery by the TA technique than for treatment by the FSA technique (Table 2). Concerning AG, oedema and pain, the linear mixed model resulted in a statistically significant difference between the TA and FSA (*p* = 0.003; *p* < 0.01; and *p* = 0.018, respectively). Conversely, the model did not show a difference (*p* = 0.322) between the two methods if PPD was taken into account. For the GLM method, degrees of liberty and F were 8 and 5.4, 5 and 16.4, and 5 and 3.0, respectively. The FSA procedure was statistically faster than the TA procedure (17 min and 8 s (±6 s) vs. 28 min and 6 s (±4 s), respectively) (*p* < 0.05).

It is notable that after 8 weeks there was hardly any sign of the surgery visible on either side (Figure 2f). Lastly, concerning the statistical power of the sample size, the analysis of the primary variables of our data confirms that 12 patients and 24 teeth allow the desired power of 80% for attached gingiva and more than 90% for oedema.

## 4. Discussion

Surgical removal of impacted Ms3 is an operation that can be associated with clinical outcomes and periodontal problems [5,20,21,22] Recent research by Øyri et al. [3], however, showed that, if correctly programmed and performed, the risk associated with M3 extraction can be quite well administrated and reduced. Therefore, the purpose of our study was to investigate the suitability of a novel flapless surgical design for lower M3 removal. Basically, we compared the novel FSA with the TA method in terms of pain, oedema, attached gingiva, and pocket probing depth recovery, as well as the average duration of the surgery. Our data showed the FSA undoubtedly supports reduction in oedema. About half our patients treated with the TA had oedema 48 h after surgery, against only the one 30-year-old patient with oedema after the FSA; the difference drastically increased after 72 h. Pain also seems to be reduced with statistical significance at 48 h and 72 h after the FSA treatment compared to the TA. Our FSA appears to make a statistically significant improvement with respect to the TA concerning the amount of affected gingiva, while the pocket probing depth prevalently restored the pre-operative conditions and, in some cases, improved irrespective of the approach used. However, one 30-year-old male patient treated with the FSA experienced a bad post-operative recovery and evident deterioration of the pocket probing depth.

To go into discussion, it is known that patients younger than 30 years of age have better full recovery of their M3 injury [23,24] and the risk of complications and postoperative morbidity [22], as well as the incidence of transitory lip sensitivity [25], in patients younger than 25 years old are reported to be lower compared with older patients. Therefore, our data agree well with many studies reporting the age of 25 years as a critical time, after which complications increase more rapidly. However, a 30-year-old female patient showed, in our study, a perfect FSA post-surgery recovery. Some authors [3] have concluded that female patients older than 21 years should be informed that M3 surgery reduces oral function and that pain recovery will be prolonged compared with younger patients and men. Nevertheless, our data are too limited to have a reliable conclusion about this concern.

Regarding swelling and pain, which are the most common symptoms that affect patients’ quality of life after M3 surgery [20,26], the improved post-operative recovery derived from the novel FSA with respect to the TA probably lies in the flap management. In the novel approach, a partial-thickness flap is removed and the wound heals through open follow-up care; sutures are absent. Indeed, a recent study highlighted that surgical drainage has a positive effect on postoperative reactions after the removal of Ms3 [27,28], and in a pioneering work, Pajarola and Sailer [29] concluded that an open wound healing method after surgical removal of Ms3 is advocated. Lastly, the possible clinical feasibility of using the FSA is also validated by a considerable reduction in surgery time, which improves the patient’s comfort.

Undoubtedly, our study shows some weaknesses; the sample size and the presence of patients older than 25 years old are among them. Indeed, our results on pocket probing depth recovery could have limitations because of them. The single operator can also be an additional weakness of the study, although because of the number of patients, it can represent a benefit to avoid bias.

However, our data also have strengths, such as the sample sizes with a statistical power from 0.80 to 0.90 depending on the variable considered, which support our evident results on attached gingiva, oedema, and pain, as well as the faster surgical time. Moreover, the comparison between our FSA and of the most widely used and reliable techniques for the removal of impacted Ms3 (TA) provides support to the reliability of our experimental setup. To support the correct experimental approaches, we can also report that the patients did not experience infection or alveolitis, and only one patient had a mild bleed to both the Ms3 extracted; alveolitis, infection and haemorrhage are common complications during or after M3 surgery [20]. Lastly, although, after surgery, there was an obvious difference in wound healing, recovery from both procedures tested in our work (FSA and TA) was completed in 8 weeks.

## 5. Conclusions

Within the limits of our study, the FSA could be a suitable option for improving the surgical removal of lower Ms3; class I-II and A-B of Pell and Gregory classification. The FSA showed an interesting perspective in terms of reduction of oedema, pain, and attached gingival management at the distal gingival margin of the 2M. The FSA also appears to be faster than the TA. Additional randomized controlled trial studies are necessary to confirm the reliability of our procedure and to verify its suitability in more complex Ms3 classifications.

## Figures and Tables

**Table 1:** Descriptives of recorded variables split between the traditional–envelope–flap (traditional approach (TA)) and the flapless surgical approach (FSA), expressed as mean (standard deviation). AG = attached gingiva, PPD = pocket probing depth. T0 = baseline; T1 = 8 weeks. * is for a significative difference between time T0 and T1.

Variable	TA		FSA	
	T0	T1	T0	T1
AG	3.4 (0.6)	3.2 (0.8)	2.9 (0.8)	3.3 (0.5) *
PPD	5.4 (1.9)	3.5 (1.0)	5.4 (2.1)	3.6 (1.0)

**Table 2:** Descriptives of recorded variables split between the traditional-envelope-flap (traditional approach [TA]) and the flapless surgical approach (FSA), expressed as mean (standard deviation). OED = oedema score, PS = pain score, T0 = baseline (day 1, 24 h), T1 = day 2 (48 h) and T2 = day 3 (72 h). * is for a significative difference between time T0, T1 or T2 of TA compared to its corresponding time of FSA.

Variable	TA			FSA		
	T0	T1	T2	T0	T1	T2
OED	0.0 (0.0)	0.6 * (0.5)	1.8 * (1.3)	0.0 (0.0)	0.1 (0.3)	0.1 (0.3)
PS	0.4 (1.2)	1.3 * (1.2)	0.8 * (1.3)	0.3 (0.9)	0.1 (0.3)	0.2 (0.4)

## Data Availability

Data available on request from the authors.

## Supplementary Materials

The following are available online at https://www.mdpi.com/2077-0383/10/4/593/s1, Figure S1: Flow Chart for the Experiment.
