# Supplementary figures and images for: Flapless Surgical Approach to Extract Impacted Inferior Third Molars: A Retrospective Clinical Study
Source: J Clin Med. 2021 Feb 4;10(4):593. doi: 10.3390/jcm10040593 (PMC7914559; doi:10.3390/jcm10040593)

**Figure S1: Flow Chart for the Experiment**

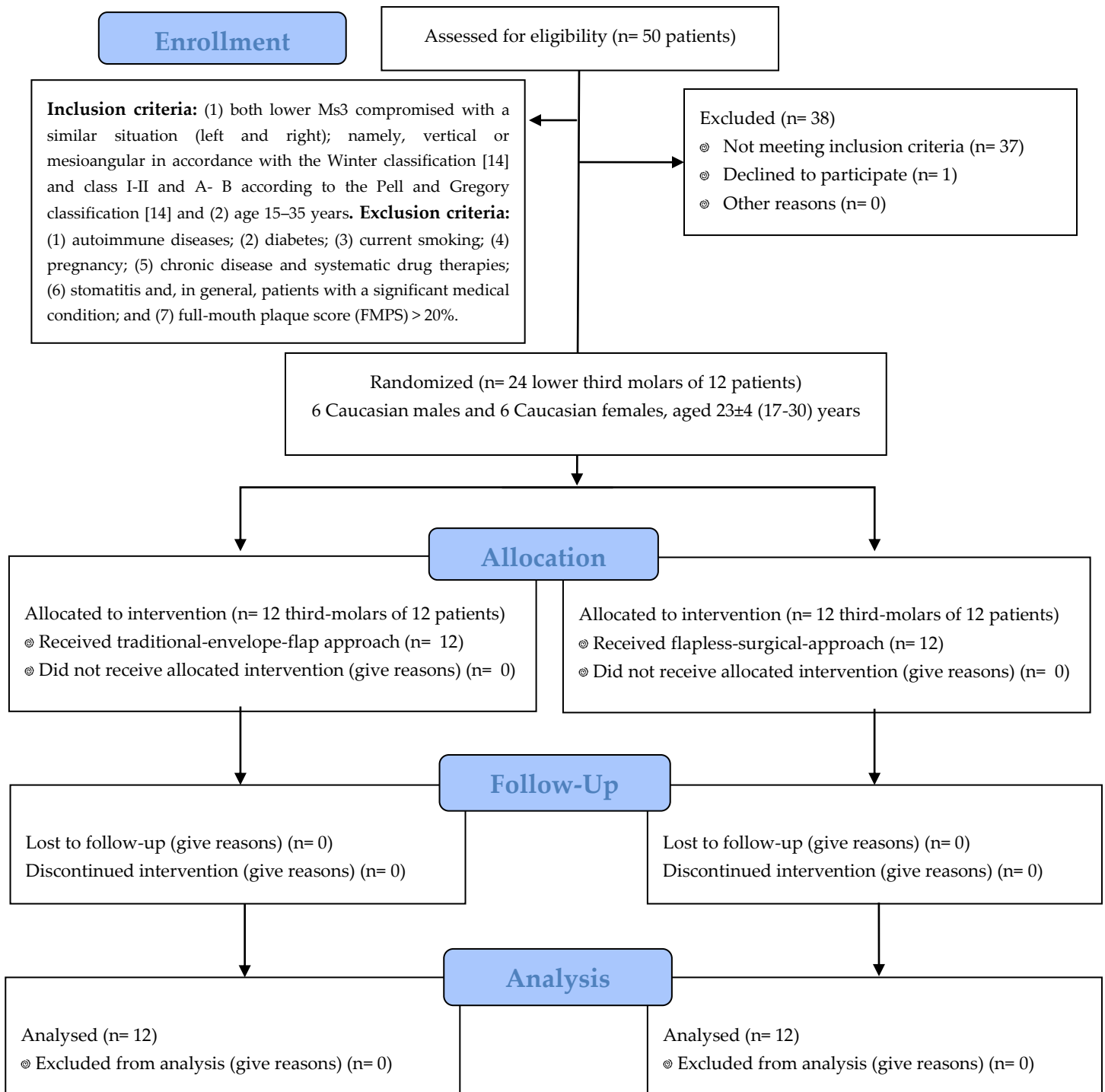

Supplement: Supplementary file 1 [file jcm-10-00593-s001.pdf]
